# Development and Validation of Prediction Models for the 5-year Risk of Type 2 Diabetes in a Japanese Population: Japan Public Health Center-based Prospective (JPHC) Diabetes Study

**DOI:** 10.2188/jea.JE20220329

**Published:** 2024-04-05

**Authors:** Juan Xu, Atsushi Goto, Maki Konishi, Masayuki Kato, Tetsuya Mizoue, Yasuo Terauchi, Shoichiro Tsugane, Norie Sawada, Mitsuhiko Noda

**Affiliations:** 1 Department of Endocrinology and Metabolism, Graduate School of Medicine, Yokohama City University, Yokohama, Japan; 2 Department of Health Data Science, Graduate School of Data Science, Yokohama City University, Yokohama, Japan; 3 Department of Epidemiology and Prevention, Center for Clinical Sciences, National Center for Global Health and Medicine, Tokyo, Japan; 4 Health Management Center and Diagnostic Imaging Center, Toranomon Hospital, Tokyo, Japan; 5 Division of Cohort Research, National Cancer Center Institute for Cancer Control, Tokyo, Japan; 6 National Institute of Health and Nutrition, National Institutes of Biomedical Innovation, Health and Nutrition, Tokyo, Japan; 7 Department of Diabetes, Metabolism and Endocrinology, Ichikawa Hospital, International University of Health and Welfare, Chiba, Japan

**Keywords:** diabetes, risk score, prediction model, Japanese population, Japan Public Health Center-based Prospective (JPHC) Study

## Abstract

**Background:**

This study aimed to develop models to predict the 5-year incidence of type 2 diabetes mellitus (T2DM) in a Japanese population and validate them externally in an independent Japanese population.

**Methods:**

Data from 10,986 participants (aged 46–75 years) in the development cohort of the Japan Public Health Center-based Prospective Diabetes Study and 11,345 participants (aged 46–75 years) in the validation cohort of the Japan Epidemiology Collaboration on Occupational Health Study were used to develop and validate the risk scores in logistic regression models.

**Results:**

We considered non-invasive (sex, body mass index, family history of diabetes mellitus, and diastolic blood pressure) and invasive (glycated hemoglobin [HbA1c] and fasting plasma glucose [FPG]) predictors to predict the 5-year probability of incident diabetes. The area under the receiver operating characteristic curve was 0.643 for the non-invasive risk model, 0.786 for the invasive risk model with HbA1c but not FPG, and 0.845 for the invasive risk model with HbA1c and FPG. The optimism for the performance of all models was small by internal validation. In the internal-external cross-validation, these models tended to show similar discriminative ability across different areas. The discriminative ability of each model was confirmed using external validation datasets. The invasive risk model with only HbA1c was well-calibrated in the validation cohort.

**Conclusion:**

Our invasive risk models are expected to discriminate between high- and low-risk individuals with T2DM in a Japanese population.

## INTRODUCTION

Diabetes mellitus (DM) is a group of metabolic diseases characterized by hyperglycemia resulting from defects in insulin secretion, insulin action, or both.^1^ According to the International Diabetes Federation, the global prevalence of diabetes in 2021 was estimated to be 10.5% (537 million people) and was expected to rise to 12.2% (783 million) by 2045.^2^ Diabetes is thought to be one of the top 10 causes of adult death.^3^ In Japan, because of its aging population, the absolute number of people with diabetes is expected to substantially increase in the coming decades.^4^ Since several intervention studies in different ethnic populations have demonstrated that type 2 diabetes mellitus (T2DM) can be effectively prevented through diet and lifestyle modifications in high-risk individuals,^5^^–^^8^ identifying high-risk individuals and having them make diet and lifestyle changes is important for preventing diabetes onset.

A disease risk score is a calculated number or score that estimates the probability or rate of disease occurrence, derived from the risk factors of the disease. At present, there are several diabetes risk scores.^9^^–^^13^ However, the substantial differences in diabetes incidence among ethnic groups^14^^,^^15^ impact the performance of each model.^16^ Although there are at least six diabetes risk prediction models for the Japanese population,^17^^–^^22^ none are based on a general population across multiple areas in Japan. Although invasive risk scores are likely to have better predictive performance, non-invasive risk scores may be useful because they are less expensive and more convenient than invasive risk scores in large-scale screening.

Therefore, we aimed to develop regression models that used non-invasive and invasive predictors to predict the 5-year incidence of diabetes in a Japanese population and validate them externally in an independent Japanese population.

## METHODS

### Study population

The Japan Public Health Center-based Prospective Study (JPHC Study), designed to collect evidence based on multipurpose cohort studies to benefit health maintenance and improvement approaches, was initiated in 1990 for Cohort I and in 1993 for Cohort II. It included residents of 11 public health center areas (Iwate, Akita, Nagano, Okinawa, and Tokyo Prefectures for Cohort I; Ibaraki, Niigata, Kochi, Nagasaki, Okinawa, and Osaka Prefectures for Cohort II), aged 40–69 years at each baseline survey. Participants in this analysis underwent annual health checkups, completed self-administered questionnaire surveys, and provided blood samples. Specific details of the study design have been published previously.^23^

The JPHC Diabetes Study started in 1998–1999 for Cohort II (residents of the Osaka Prefecture were excluded because the health checkup schedule was different from those of the other areas) and in 2000–2001 for Cohort I. In the baseline surveys, participants in Cohort I were 51–70 years old and 46–75 years old in Cohort II. A self-administered questionnaire, given during health checkups, collected data regarding family history of diabetes, previous diabetes examination results, any diagnosis of diabetes by a physician, current diabetes medications, signs of diabetic complications, a brief history of changes in body weight, time spent walking, and childbirth history.^24^ The 5-year follow-up survey was performed in the same way in 2003–2004 for Cohort II and in 2005–2006 for Cohort I.

**Figure 1.**
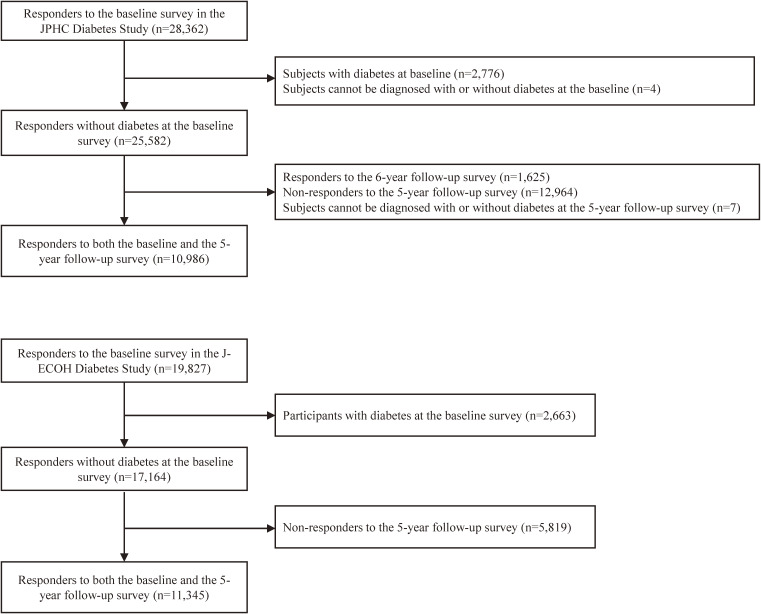
Participant selection flow diagram for the development and validation cohorts

Among 28,362 adults enrolled in the baseline survey of this study, 10,986 (39%) were included in the final analysis. As shown in Figure 1, participants with diabetes (*n* = 2,776) and those whose diabetes status could not be determined (*n* = 4) at the baseline survey were excluded. Then, participants who responded to the 6-year follow-up survey but not to the 5-year follow-up survey (*n* = 1,625) and those who did not respond to the 5-year follow-up survey (*n* = 12,964) were excluded. Finally, participants who could not be diagnosed as being either diabetic or non-diabetic (*n* = 7) at the 5-year follow-up survey were excluded. The remaining 10,986 participants were included in the analysis to develop a prediction model.

The Japan Epidemiology Collaboration on Occupational Health (J-ECOH) Study is an ongoing multi-center epidemiologic study conducted on workers from 12 companies spanning various industries; details of the study design have been published elsewhere.^20^ For the present external validation, we retrieved data from one participating company that provided health checkup data, including a family history of diabetes, and defined an analytic cohort comprising individuals who had received health checkups in the fiscal year 2013 (baseline). As described elsewhere,^25^ study participants in the J-ECOH study were asked to select up to three activities from a list of 20 activities and the frequency (times per month) and duration (minutes per occasion) for each activity. Leisure-time physical activity (minutes per month) was computed by summing up the duration of activities reported by each participant. A total of 19,827 participants aged 46–75 years underwent a baseline checkup and had no missing data necessary for the validation analysis. Of these, individuals with diabetes at baseline (*n* = 2,663) and non-attendants to the 5-year health checkup in the fiscal year 2018 (*n* = 5,819) were excluded. Finally, 11,345 (57%) were used to validate the prediction models (Figure 1).

All participants provided written informed consent. The JPHC Study was approved by the ethics committees of Yokohama City University and the National Cancer Center, Japan, and was also approved by the ethics committee of the National Center for Global Health and Medicine, Japan. The J-ECOH study was approved by the Ethics Committee of the National Center for Global Health and Medicine, Japan.

### Predictors

Based on previous literature, we selected 16 potential diabetes predictors (non-invasive predictors: age, sex, body mass index [BMI], time spent walking, family history of DM, systolic blood pressure [SBP], and diastolic blood pressure [DBP]; levels of invasive predictors: alanine aminotransferase [ALT], aspartate aminotransferase [AST], γ-glutamyl transferase [GGT], high-density lipoprotein [HDL], total cholesterol [TC], triglyceride [TG], estimated glomerular filtration rate [eGFR], fasting plasma glucose [FPG], and glycated hemoglobin [HbA1c]). All these factors were associated with the development of T2DM in previous studies.^26^^–^^34^

Data on age, height, weight, time spent walking, and family history of DM were acquired from the questionnaire; BMI was calculated as the weight in kilograms divided by the squared height in meters. The participants were classified into four levels based on the time spent walking: walking time <0.5, 0.5 to <1, 1 to <2, or ≥2 hours per day. A family history of diabetes was defined as the presence of diabetes in first-degree relatives. Blood pressure measurements were recorded during the health checkups.

When collecting blood samples, participants were not required to fast. Since fasting status has a great influence on TG levels, this parameter was excluded from our analysis. eGFR (mL/min/1.73 m^2^) was calculated using the formula: = 194 × serum creatinine^−1.094^ × age^−0.287^ × 0.739 (if female).^35^ The recorded HbA1c level (expressed per the Japan Diabetes Society [JDS]) was converted to the National Glycohemoglobin Standardization Program (NGSP) equivalent using the following formula: HbA1c (%) = 1.02 × HbA1c (JDS) (%) + 0.25%.^36^

### Primary outcome measures

The diagnostic criteria for DM were as follows: (1) HbA1c value ≥6.5%, (2) FPG value ≥126 mg/dL, (3) random plasma glucose level ≥200 mg/dL, (4) physician-diagnosed DM (self-reported), or (5) undergoing any kind of diabetes treatment, including diet or exercise interventions (self-reported). These diagnostic criteria were used to exclude patients with diabetes at baseline and to confirm the number of patients diagnosed with diabetes at the 5-year follow-up in both the JPHC and J-ECOH studies. It was previously shown that 94% of self-reported diabetes cases were confirmed using medical reports in a subsample of the JPHC Study participants.^37^

### Statistical analysis

After the multiple imputations as described later, logistic regression models were used to develop prediction models for diabetes incidence and to estimate β coefficients, odds ratios (ORs), and 95% confidence intervals (CIs). First, we examined all variables in the univariate regression model. We used a multiple logistic regression model with backward variable selection (fastbw function from the rms package) to determine significant variables in each multiple imputed dataset and in each JPHC Diabetes Study area. Predictors selected in more than 50% of the multiple imputed datasets among >50% of the areas were included in the final models.^38^ Model 1 considered all non-invasive risk factors as potential predictors; model 2 considered all non-invasive and invasive predictors, except FPG; and model 3 considered all variables. Because the proportion of available FPG values was low, a model with FPG could produce unstable estimates because of missing data. Therefore, we developed models 2 and 3 separately, although we imputed the FPG values using the multiple imputation method.

We used the rcorr function from the Hmisc package to assess multicollinearity, which suggested that the predictors did not strongly correlate with each other. We also examined missing values for several predictors. Assuming that the probability of missing data is determined only by the observed data (ie, missing at the random condition), we used the multiple imputations by chained equations (MICE) algorithm^39^ to impute the missing data. One hundred datasets were created based on the known information to obtain different imputed values.

Among the continuous predictors, age, DBP, eGFR, and TC levels tended to be linearly associated, whereas the remaining variables were more likely to be non-linearly associated with diabetes incidence (predictors selected in the final model are shown in eFigure 1), after assessing non-linearity using restricted cubic splines (rcs function from the rms package) and Akaike’s information criterion (AIC function from the stats package). The rcs function was used to fit the nonlinear regression models by setting up special attributes (such as knots and nonlinear term indicators). The AIC evaluates how well a model fits the data (a smaller value of AIC is better).^40^ Pooled β coefficients were estimated over the imputed datasets (fit.mult.impute function from the Hmisc package). All analyses were performed using R, version 4.2.0 (R Foundation for Statistical Computing, Vienna, Austria).^41^

### Model validation

The final models were developed in the entire sample (eight areas) and evaluated via an internal validation of the JPHC Study dataset. The J-ECOH Study dataset was used for external validation. For the internal validation, we assessed the discrimination of the prediction models by calculating the area under the receiver operator characteristic (ROC) curve (AUC; also known as C-statistic)^40^^,^^42^ using the roc function from the pROC package. Bootstrapping was used to quantify the optimism of our prediction models and to obtain optimism-corrected performance estimates (the number of bootstrap iterations was 1,000). Optimism-corrected performance was calculated as optimism-corrected performance = apparent performance in the original sample − optimism, where optimism = bootstrap performance − test performance).^42^ An AUC value of 0.5 indicates that the model is no better than random chance, while a value of 1 indicates that the model perfectly distinguishes cases and non-cases. We assessed the calibration (the agreement of observed outcomes with the predicted risk) of the prediction models by creating calibration plots using the val.prob.ci.2 function from the CalibrationCurves package. Apparent AUCs and calibration plots were estimated using a stacked dataset that stacks the 100 imputed data sets into a single data set.^42^ Optimism-corrected AUCs were estimated within each imputed data set and averaged over 100 imputed data sets to obtain summary results.^42^

In the absence of a sufficiently large sample size, a random split sample approach or a non-random split sample approach is likely to provide unstable validation results. Therefore, to validate prediction models in different settings, we performed the internal-external cross-validation in the JPHC Diabetes Study (eFigure 2), as recommended by Steyerberg and Harrell.^42^^,^^43^ For the internal-external cross-validation, the model development was performed in seven areas by sequentially dropping one area at a time. Then, the models were validated in the omitted area by calculating AUC using the roc function from the pROC package.

For external validation, the discrimination and calibration performances of the developed models also used AUCs (roc function from the pROC package) and calibration plots (val.prob.ci.2 function from the CalibrationCurves package). In addition, to adjust the predicted risks for the validation cohort, we estimated the correction factor by using the function odds_adjust from the predtools package.

All analyses for model validation were conducted in each imputed dataset, and validation parameters were averaged to obtain pooled results.

To understand the impact on participants who did not participate in the follow-up survey, sensitivity analyses were also performed for the JPHC Diabetes Study and the J-ECOH Study. Sensitivity analyses included all participants without diabetes at baseline. MICE was also used to impute missing data and 100 datasets were created based on known information to obtain different imputed values. Since people who did not participate in the 5-year follow-up survey could not determine whether they had diabetes, we counted the status of the patients in 100 datasets after imputation. If they were considered to have diabetes in more than 50 datasets, they were diagnosed with diabetes, otherwise, they were not. The average of probability was used to create the calibration plot.

### Model presentation

The models were presented as formula based on the logistic regression coefficients. Thereafter, the risk score was calculated using an Excel spreadsheet (Microsoft Corp., Redmond, WA, USA) created according to the formula (eTable 1). In addition, the study followed the Transparent Reporting of a multivariable prediction model for Individual Prognosis Or Diagnosis (TRIPOD) statement^44^ to improve the transparency and quality of reporting of these prediction models.

## RESULTS

The characteristics of the JPHC Study participants are presented in Table 1 and eTable 2. At the 5-year follow-up, 707 (6.4%) new diabetes cases were recorded. The median age was 63 years, and the number of women was 7,377 (67.1%). People tended to exercise more than 2 hours a day (43.7%) rather than less than half an hour (12.6%). Approximately 11.2% of the participants had a family history of diabetes. Missing values were observed for 12 predictors in the derivation cohort. FPG was the variable with the most missing values in the data set, 7,131 (64.9%). The mice package was used to perform multiple imputations for the missing values. In total, 8,896 of the required 164,790 values (5.4%) were needed to impute for the final analysis.

**Table 1.** Characteristics of participants in the JPHC Diabetes Study and the J-ECOH Study^a^

Characteristic^a^	JPHC Diabetes Study (*n* = 10,986)		Characteristic^a^	J-ECOH Study (*n* = 11,345)	
	
	Value^b^	Missing values, *n* (%)		Value^b^	Missing values, *n* (%)
Age, years	63 (57–67)	0	Age, years	51 (48–54)	0
Women	7,377 (67.1%)	0	Women	1,773 (15.6%)	0
BMI, kg/m^2^	23.5 (21.5–25.6)	23 (0.2)	BMI, kg/m^2^	23.2 (21.4–25.3)	0
Walking time, (hours per day)			Leisure-time physical activity, minutes per month	0 (0–84)	391 (3.4)
<0.5 hours	1,379 (12.6%)	130 (1.2)			
0.5 hours to <1 hour	2,322 (21.1%)				
1 hour to <2 hours	2,349 (21.4%)				
≥2 hours	4,806 (43.7%)				
Family history of diabetes	1,225 (11.2%)	0	Family history of diabetes	1,996 (17.6%)	0
SBP, mm Hg	130 (119–140)	6 (0.1)	SBP, mm Hg	122 (113–130)	0
DBP, mm Hg	78 (70–84)	6 (0.1)	DBP, mm Hg	79 (72–84)	0
HDL, mg/dL	57 (48–67)	1 (0.0)	HDL, mg/dL	55 (46–65)	0
TC, mg/dL	207 (186–230)	1 (0.0)	TC, mg/dL	201 (181–221)	16 (0.1)
FPG, mg/dL	93 (88–100)	7,131 (64.9)	FPG, mg/dL	98 (92–105)	0
HbA1c, %	5.5 (5.1–5.7)	34 (0.3)	HbA1c, %	5.5 (5.3–5.7)	0
ALT, IU/L	18 (15–24)	7 (0.1)	ALT, IU/L	21 (16–29)	0
AST, IU/L	22 (19–27)	1 (0.0)	AST, IU/L	21 (18–26)	0
GGT, IU/L	21 (15–33)	7 (0.1)	GGT, IU/L	30 (20–51)	0
eGFR, mL/min/1.73 m^2^	73.8 (63.4–82.5)	1,549 (14.1)	eGFR, mL/min/1.73 m^2^	78.8 (69.7–89.4)	5,549 (48.9)

5-year outcome	707 (6.4%)	0	5-year outcome	673 (5.9%)	0

ALT, alanine aminotransferase; AST, aspartate aminotransferase; BMI, body mass index; DBP, diastolic blood pressure; eGFR, estimated glomerular filtration rate; FPG, fasting plasma glucose; GGT, γ-glutamyl transferase; HbA1c, glycated hemoglobin; HDL, high-density lipoprotein; SBP, systolic blood pressure; TC, total cholesterol.

^a^Characteristics were collected at baseline.

^b^Continuous variables are medians (interquartile ranges) and categorical variables are numbers (percentages).

Characteristics of the J-ECOH Study participants are presented in Table 1. There were fewer women (15.6%), and approximately 17.6% of participants had a family history of diabetes in the J-ECOH study. There were 673 (5.9%) new diabetes cases at the 5-year follow-up. We also compared the baseline characteristics of participants who were not included in the final analysis of the JPHC Diabetes Study and the J-ECOH Study and found that they had similar characteristics to the analyzed participants (eTable 3).

Table 2 shows the differences in parameters between participants with and without diabetes and the relationship between risk factors and type 2 diabetes risk. There was little difference in age between participants with and without incident diabetes; however, there was a higher proportion of men among those with incident diabetes than among those without it. The risk of diabetes decreased with increased walking time. In addition, participants with incident type 2 diabetes had a family history of diabetes more frequently. For continuous variables (BMI, SBP, DBP, and the levels of ALT, AST, GGT, TC, FPG, and HbA1c), the median values were higher in the diabetes group than in the non-diabetes group. In contrast, HDL levels tended to be lower in those with incident diabetes than in those without diabetes.

**Table 2.** Distribution of study variables by DM status in the JPHC Diabetes Study

Characteristics^b^	Participants without incident DM^a^ (*n* = 10,279)	Participants with incident DM^a^ (*n* = 707)	Odds ratio (95% CI)^c,d^			

			Univariate	Model 1	Model 2	Model 3
Age,^e^ years	63 (57–67)	64 (59–68)	1.23 (1.09–1.38)	—	—	—
Sex, %						
Female	6,980 (95%)	397 (5%)	1 (ref.)	1 (ref.)	—	—
Male	3,299 (91%)	310 (9%)	1.65 (1.42–1.93)	1.74 (1.49–2.04)	—	—
BMI, kg/m^2^	23.5 (21.5–25.5)	24.5 (22.4–26.7)	1.78 (1.45–2.18)	1.73 (1.41–2.13)	—	—
Walking time,^e^ hours per day						
<0.5 hours	1,278 (93%)	101 (7%)	1.22 (0.97–1.54)	—	—	—
0.5 hours to <1 hour	2,164 (93%)	158 (7%)	1.13 (0.92–1.38)	—	—	—
1 hour to <2 hours	2,196 (93%)	153 (7%)	1.08 (0.88–1.32)	—	—	—
≥2 hours	4,514 (94%)	292 (6%)	1 (ref.)	—	—	—
Family history of diabetes, %						
Yes	1,082 (88%)	143 (12%)	2.16 (1.78–2.62)	2.26 (1.86–2.75)	1.64 (1.33–2.03)	1.56 (1.23–1.98)
No	9,197 (94%)	564 (6%)	1 (ref.)	1 (ref.)	1 (ref.)	1 (ref.)
SBP,^e^ mm Hg	130 (118–140)	134 (124–144)	1.44 (1.29–1.60)		—	—
DBP, mm Hg	78 (70–84)	80 (70–86)	1.19 (1.08–1.32)	1.04 (0.94–1.16)	—	—
HDL,^e^ mg/dL	57 (48–68)	53 (45–64)	0.60 (0.49–0.74)	—	—	—
TC,^e^ mg/dL	207 (186–229)	211 (188–232)	1.13 (1.02–1.25)	—	—	—
FPG, mg/dL	93 (88–99)	106 (97–115)	4.16 (2.83–6.10)	—	—	2.95 (1.98–4.39)
HbA1c, %	5.4 (5.1–5.7)	5.9 (5.6–6.1)	3.50 (2.91–4.22)	—	3.44 (2.86–4.13)	2.63 (2.17–3.19)
ALT,^e^ IU/L	18 (14–24)	21 (16–28)	1.58 (1.37–1.83)	—	—	—
AST,^e^ IU/L	22 (19–26)	24 (20–29)	1.54 (1.32–1.79)	—	—	—
GGT,^e^ IU/L	21 (15–32)	26 (18–43)	2.07 (1.77–2.42)	—	—	—
eGFR,^e^ mL/min/1.73 m^2^	73.8 (63.4–82.5)	73.5 (63.4–83.0)	0.98 (0.92–1.06)	—	—	—

ALT, alanine aminotransferase; AST, aspartate aminotransferase; BMI, body mass index; CI, confidence interval; DBP, diastolic blood pressure; DM, diabetes mellitus; eGFR, estimated glomerular filtration rate; FPG, fasting plasma glucose; GGT, γ-glutamyl transferase; HbA1c, glycated hemoglobin; HDL, high-density lipoprotein; ref., reference; SBP, systolic blood pressure; TC, total cholesterol.

^a^Continuous variables are shown as medians (interquartile ranges) and categorical variables as numbers (percentages) unless otherwise indicated.

^b^A backward stepwise variable selection method was used to select the variables to be included in the prediction model.

^c^Odds ratios were estimated using logistic regression models after multiple imputations. Model 1 included sex, BMI, family history of DM, and DBP. Model 2 included a family history of DM, and HbA1c. Model 3 included a family history of DM, FPG level and HbA1c.

^d^Interquartile range (0.75 vs 0.25 quantile) odds ratios are shown for continuous variables. For example, odds ratio for age compares the 3rd quartile with the 1st quartile of age. Odds ratios for categorical predictors were compared between each group and the reference group (the smallest group).

^e^Not included in each model after the backward stepwise variable selection method.

**Figure 2.**
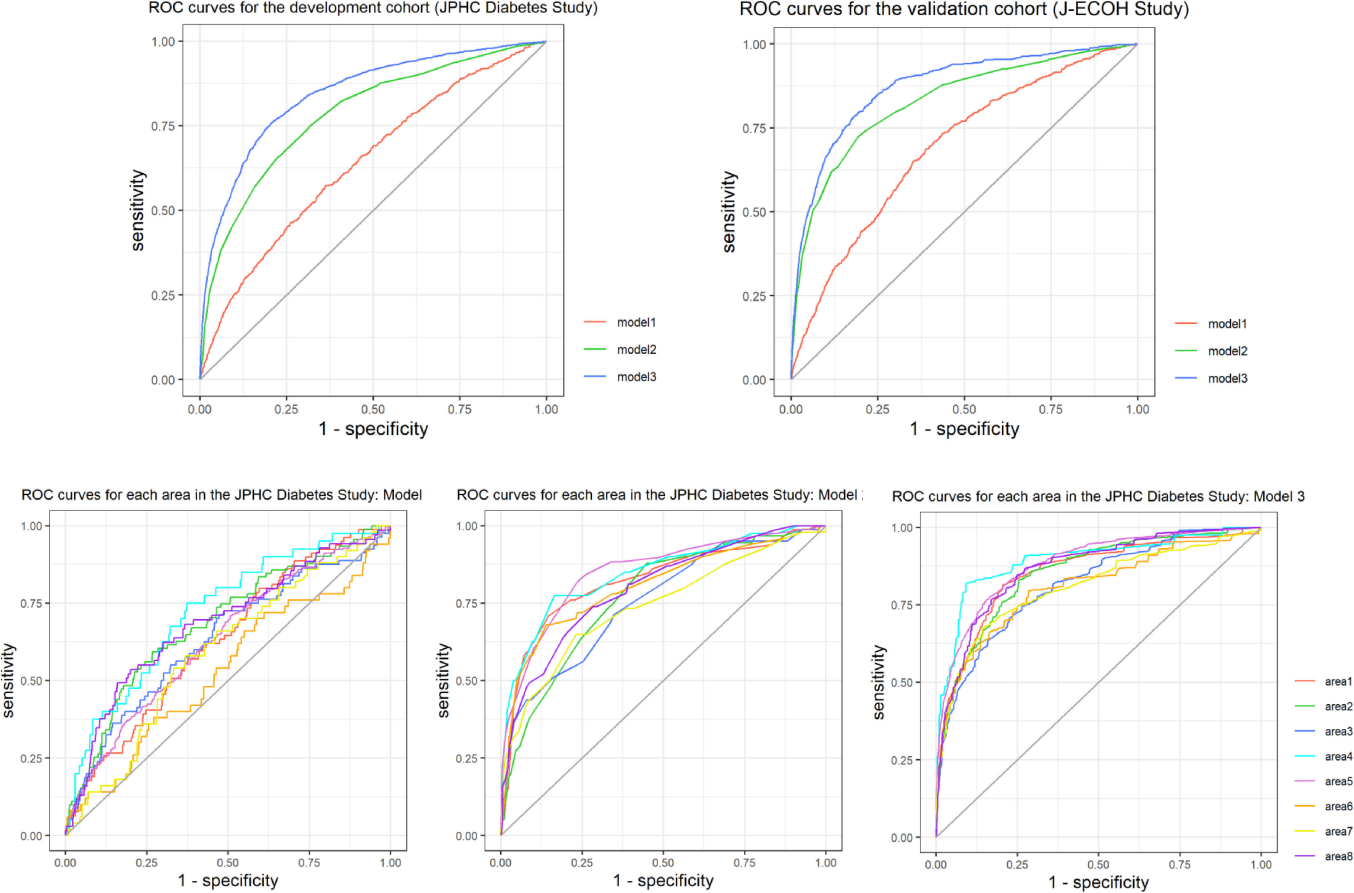
Receiver operating characteristic curves for the development and validation cohorts AUC, the area under the receiver operating characteristic (ROC) curve; BMI, body mass index; DBP, diastolic blood pressure; FPG, fasting plasma glucose; HbA1c, glycated hemoglobin. Model 1: included sex, BMI, a family history of DM, and DBP. Model 2: included a family history of DM and HbA1c Model 3: included a family history of DM, HbA1c, and FPG C-statistic (AUC): in the JPHC Diabetes Study, Model 1 = 0.643, Model 2 = 0.786, and Model 3 = 0.845; after optimism correction, the AUCs decreased to 0.639, 0.785, and 0.844, respectively. The number of bootstrap iterations was 1000. After internal-external cross-validation, the AUCs of each area in Model 1 = 0.629, 0.688, 0.634, 0.723, 0.633, 0.532, 0.595, and 0.686, respectively; the AUCs of each area in Model 2 = 0.823, 0.772, 0.754, 0.846, 0.851, 0.806, 0.742, and 0.798, respectively; the AUCs of each area in Model 3 = 0.855, 0.853, 0.817, 0.895, 0.884, 0.807, 0.809, and 0.868, respectively. The AUCs in the J-ECOH Study were 0.692, 0.831, and 0.874 in Models 1, 2, and 3, respectively.

Finally, sex, BMI, family history of DM, and DBP were selected for model 1, family history of DM and HbA1c for model 2, and family history of DM, HbA1c, and FPG for model 3. For internal-external cross-validation, the AUCs of model 1 ranged from 0.532 to 0.723, the AUCs of model 2 ranged from 0.742 to 0.851, and the AUCs of model 3 ranged from 0.807 to 0.895 (Figure 2). For the internal validation of the final models, the model performance is shown in Figure 2. The AUC of model 1 was 0.643, that of model 2 yielded an AUC of 0.786, and that of model 3 had an AUC of 0.845. After bootstrap optimism correction, the AUCs slightly decreased to 0.639, 0.785, and 0.844, respectively. The discriminative ability of each model was confirmed in the J-ECOH Study; the AUCs were 0.692, 0.831, and 0.874 in models 1, 2, and 3, respectively.

**Figure 3.**
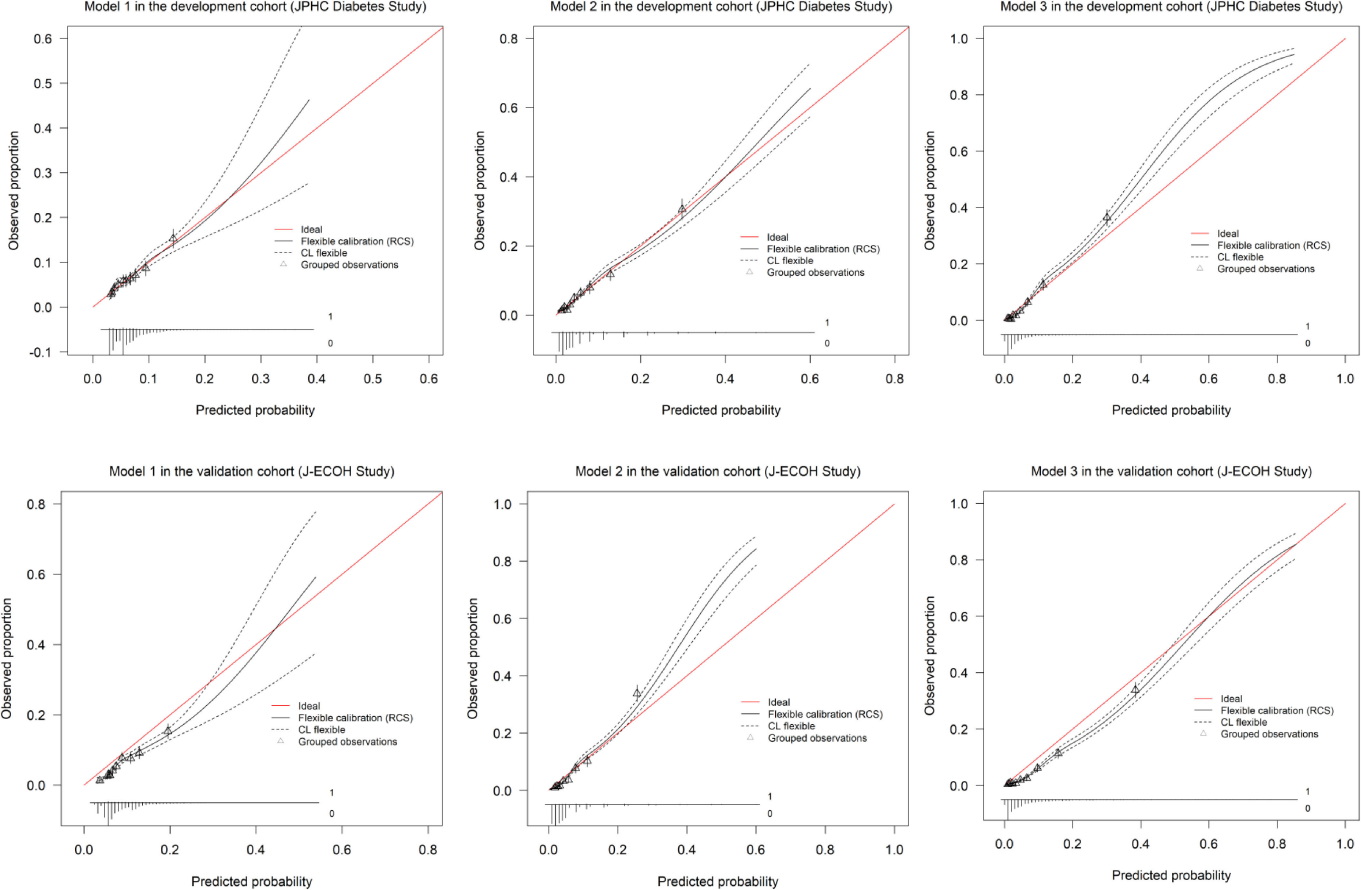
Calibration plots to show relations between predicted and observed probabilities in the development and validation cohorts BMI, body mass index; DBP, diastolic blood pressure; FPG, fasting plasma glucose; HbA1c, glycated hemoglobin. Model 1: included sex, BMI, a family history of DM, and DBP. Model 2: included a family history of DM and HbA1c Model 3: included a family history of DM, HbA1c, and FPG Calibration plots were created to graphically assess the agreement of the mean observed risk with the mean predicted risk according to the deciles of the predicted risk. Ideal: ideal line for the prediction model. Flexible calibration (RCS): “RCS” generates a flexible calibration curve based on restricted cubic splines. CL flexible: 95% confidence limits for the flexible calibration curve with dashed lines. Grouped observations: mean predicted probability and observed proportion of diabetes incidence in each of the deciles (ten groups of equal size).

The calibration curves (Figure 3) indicated that the predicted and empirical probabilities were close to each other, indicating that the prediction models fitted the data well in the development cohort. As shown in Figure 3, the probability of diabetes in high-risk participants was overestimated in models 1 and 3 in the validation cohort. The extent of agreement between the observed outcomes and predicted risk in model 2 was better than that in models 1 and 3 in the validation cohort.

The predictive performance did not materially change when a family history of diabetes was defined as the presence of diabetes in a family member, regardless of the degree of the relationship (eTable 4; eFigure 3). In addition, the calibration plots in the validation cohort remained unchanged after the intercept adjustments (eFigure 4). After a sensitivity analysis that included participants who did not participate in the follow-up survey, the AUCs in the JPHC Diabetes Study changed to 0.631, 0.764, and 0.848, and those in the J-ECOH Study changed to 0.676, 0.834, and 0.874 in models 1, 2, and 3, respectively (eFigure 5). The calibration performance did not improve in the sensitivity analysis, as shown in eFigure 6.

Table 3 shows the content of the Excel spreadsheet used to obtain approximate predictions for the individuals. Using the medians for continuous predictors and the category with more participants for categorical variables, we calculated the average risk probability of DM to be 3.94% in model 1, 3.32% in model 2, and 1.54% in model 3. Here, we provide an example using model 2 to show how to obtain DM risk probability. A male with a family history of diabetes demonstrated a BMI of 25 kg/m^2^, a diastolic blood pressure of 80 mm Hg, and an HbA1c of 6%. By entering these data into Excel, the risk of DM was estimated to be 23.89%.

**Table 3.** Prediction Model and Calculation Table

Predictors^a^	Variables	Units	Coefficient			Average values^c^	Coefficient × Average values			Your patient (Example using Model 2)^d^	
	
			Model 1	Model 2	Model 3		Model 1	Model 2	Model 3		
Constant	Intercept	—	−1.47	−7.96	−4.11	1	−1.47	−7.96	−4.11	1	−7.96
Sex	Female	0/1	−0.56			1	−0.56	—	—	**0**	—
BMI	BMI	kg/m^2^	−0.08	—	—	23.50	−1.83	—	—	**25**	—
	(BMI-19.0)^3^+		0.00	—	—	91.13	0.45	—	—	216.00	—
	(BMI-22.4)^3^+		−0.01	—	—	1.33	−0.02	—	—	17.58	—
	(BMI-24.7)^3^+		0.01	—	—	0.00	0.00	—	—	0.03	—
	(BMI-28.9)^3^+		−0.00	—	—	0.00	0.00	—	—	0.00	—
Family history of DM	Family history of DM	0/1	0.82	0.50	0.45	0	0.00	0.00	0.00	**1**	0.50
DBP^b^	DBP	mm Hg	0.00	—	—	78	0.24	—	—	**80**	—
HbA1c^b^	HbA1c	%	—	0.77	0.44	5.5	—	4.24	2.43	**6.0**	4.63
	(HbA1c-4.9)^3^+		—	1.59	1.44	0.2	—	0.34	0.31	1.33	2.11
	(HbA1c-5.5)^3^+		—	−3.49	−3.17	0.0	—	0.00	0.00	0.13	−0.44
	(HbA1c-6.0)^3^+		—	1.90	1.73	0.0	—	0.00	0.00	0.00	0.00
FPG^b^	FPG	mg/dL	—	—	−0.03	93	—	—	−3.02	**100**	—
	(FPG-81)^3^+		—	—	0.00	1,728.0	—	—	0.07	6,859.00	—
	(FPG-88)^3^+		—	—	0.00	125.00	—	—	0.16	1,728.00	—
	(FPG-93)^3^+		—	—	−0.00	0.00	—	—	0.00	343.00	—
	(FPG-99)^3^+		—	—	0.00	0.00	—	—	0.00	1.00	—
	(FPG-112)^3^+		—	—	−0.00	0.00	—	—	0.00	0.00	—

						Probability	3.94%	3.32%	1.54%		23.89%

BMI, body mass index; DBP, diastolic blood pressure; DM, diabetes mellitus; FPG, fasting plasma glucose; HbA1c, glycated hemoglobin.

^a^Variables were selected using the backward stepwise method, and multiple imputations by chained equations (MICE) method was used to handle missing data.

^b^Knots were placed at the 10th, 50th, and 90th percentiles for HbA1c; at the 5th, 35th, 65th, and 95th percentiles for BMI, and at the 5th, 27.5th, 50th, 72.5th, and 95th percentiles for FPG.

^c^To calculate the average risk probability of the DM. The medians were used for continuous predictors. The category with more participants were used for categorical variables.

^d^An example is provided. A male with a family history of diabetes, diastolic blood pressure of 80 mm Hg, BMI of 25 kg/m^2^, and HbA1c level of 6.0%.

After pooling the coefficients in the final multivariable model, the formula for the five-year incidence of type 2 diabetes can be summarized as 1/[1 + exp(−L)], where L in Model 1 = −1.4677114 − 0.55636706 × [Sex = “female”] − 0.077979787 × BMI + 0.0048939561 × (BMI − 19.0)^3^ − 0.014293364 × (BMI − 22.4)^3^ + 0.010584929 × (BMI − 24.7)^3^ − 0.0011855209 × (BMI − 28.9)^3^ + 0.81638492 × [Family history of diabetes = “YES”] + 0.0030199043 × DBP; where L in Model 2 = −7.9560656 + 0.49588037 × [Family history of diabetes = “YES”] + 0.77107227 × HbA1c + 1.5861765 × (HbA1c − 4.9)^3^ − 3.4895883 × (HbA1c − 5.5)^3^ + 1.9034118 × (HbA1c − 6.0)^3^; where L in Model 3 = −4.1097962 + 0.44533254 × [Family history of diabetes = “YES”] + 0.44201803 × HbA1c + 1.4426444 × (HbA1c − 4.9)^3^ − 3.1738177 × (HbA1c − 5.5)^3^ + 1.7311733 × (HbA1c − 6.0)^3^ − 0.032485574 × FPG + 0.000040103209 × (FPG − 81)^3^ + 0.0012713229 × (FPG − 88)^3^ − 0.0028839757 × (FPG − 93)^3^ + 0.001772353 × (FPG − 99)^3^ − 0.00019980342 × (FPG − 112)^3^.

Notes: in L,

1. Square brackets [c] = 1 if the participant falls into category c; [c] = 0 otherwise.

2. Round brackets indicate (x)_+_ = x if x > 0, and (x)_+_ = 0 otherwise.

3. Measurement units: BMI (kg/m^2^), DBP (mm Hg), HbA1c (%), and FPG (mg/dL).

## DISCUSSION

In this study, we developed three models to predict the risk of DM. All models showed good discrimination and calibration in internal validations. The internal-external cross-validation indicated that these models showed similar discriminative ability across eight areas. To the best of our knowledge, this is the first diabetes risk score developed and validated using a nationwide population in Japan to predict the 5-year incidence of type 2 diabetes. For the non-invasive model, sex, BMI, family history of diabetes, and DBP were used to create a non-invasive prediction model that showed good predictive ability (AUC = 0.643) for the 5-year incidence of type 2 diabetes. The risk models that included HbA1c showed better predictive ability, with an AUC of 0.786, and the predictive model performed best when both FPG and HbA1c levels were included (AUC = 0.845), consistent with previous studies.^18^^–^^21^ Although the AUC values decreased after optimism correction, all remained reliable, as also observed in the internal-external cross-validation and external validation cohort. The AUC values were higher in the J-ECOH Study than in the JPHC Diabetes Study, indicating that the developed models were generally good at discrimination. For the calibration performance, however, calibration plots of models 1 and 3 were poor in the validation cohort. This indicates that the predicted probabilities overestimated the observed probabilities in the validation cohort. In comparison, model 2 was well-calibrated in the J-ECOH Study. Since model 2 tended to underestimate the observed probability in the highest decile of the predicted probability in the J-ECOH Study, the model should be used with caution, especially for those with a high predicted probability.

Several earlier studies developed diabetes prediction models for Japanese populations,^17^^–^^22^ including the earliest known diabetes risk score model that was published in 2008 for residents of the Ibaraki Prefecture.^17^ The model included BMI, blood glucose level, SBP, treatment for hypertension, TG levels, and smoking habits as predictors; however, it did not provide the AUC value. The Hisayama Study included 1,935 participants in the development model and 1,147 in the validation model. However, all the participants were residents of a rural town, suggesting limited study generalizability.^18^ Two risk models were established in the Hisayama Study. Age, sex, family history of diabetes, abdominal circumference, BMI, hypertension, regular exercise, and current smoking were included in the noninvasive risk model, with an AUC of 0.700, which increased to 0.772 when FPG levels were added. The participants in the Toranomon Hospital Health Management Center Study 6 mainly involved apparently healthy Japanese government employees^19^; it included four risk scores. The AUC of the model that included age, sex, family history of diabetes, current smoking, and BMI was 0.708, which increased to 0.836 when the FPG level was added, 0.837 when HbA1c was included, and 0.887 when both FPG and HbA1c levels were added. In the Japan Epidemiology Collaboration on Occupational Health Study (J-ECOH Study),^20^^,^^21^ most participants were workers in large companies, and the risk predictors did not include a family history of diabetes. Predicted probabilities of DM at 3 years and 7 years were created using age, sex, smoking status, abdominal obesity, BMI, and hypertension status in the basic model or by adding FPG or HbA1c levels or adding both FPG and HbA1c levels. The AUC values ranged from 0.717 to 0.893 for the 3-year incidence of DM and from 0.73 to 0.89 for the 7-year incidence of DM. The Aizawa Hospital Study^22^ included individuals who underwent general health examinations at the Health Center of Aizawa Hospital (development cohort, 2,080 individuals; validation cohort, 2,079 individuals).

Compared with these previous studies, we developed the model based on a population across multiple areas in Japan. Our models provided AUCs (unlike the Ibaraki Prefectural Health Study), included a family history of DM (unlike the J-ECOH Study), and were not limited to one region or occupation (unlike all the studies mentioned before). Therefore, we believe that our models are more representative of a Japanese population. We confirmed the validity of our prediction models with internal validation using bootstrapping and internal-external cross-validation in the JPHC Diabetes Study. These procedures are recommended by Steyerberg and Harrell.^42^^,^^43^ In addition, we fully utilized the information of continuous variables such as HbA1c or FPG using the cubic spline function to model potential nonlinear relations between variables and to avoid information loss. Finally, our models showed good performance in distinguishing between individuals with and without the risk of developing diabetes.

There are several possible explanations as to why the population of the J-ECOH study did not present good calibration performance. As shown in Table 1, the study participants of the J-ECOH study were younger (median age: 51 vs 63 years) and tended to have lower SBP (median: 122 vs 130 mm Hg) than those in the JPHC Diabetes Study. These factors are established risk factors for type 2 diabetes and these were not included in our prediction models, which may have affected the calibration performance.

Our study had several limitations. First, approximately 51% (12,964/25,582) of the participants without diabetes in the JPHC Diabetes Study and 34% (5,819/17,164) of the participants without diabetes in the J-ECOH Study participated in the baseline survey but did not visit the 5-year follow-up survey, potentially causing selection bias. However, when we included those who did not complete the 5-year follow-up survey and imputed the outcomes using the MICE, the results did not materially change (eFigure 5). Second, we did not conduct oral glucose tolerance tests to define the incidence of type 2 diabetes, possibly underestimating the incidence.^24^ Furthermore, although our internal validation via bootstrapping did not indicate any severe optimism, some optimism may exist because our bootstrapping procedure could not incorporate the uncertainty of the model selection and variable selection. In addition, we used the dataset from 20 years ago to create the prediction model, which may not be as accurate as data collected more recently. Finally, although our previous findings^45^ suggested that adding a genetic risk score might provide incremental model predictive performance, we did not include the genetic risk score in this study.

In conclusion, 5-year models for predicting the incidence of type 2 diabetes, with high discrimination and calibration, were developed and validated in this population-based study among a Japanese population. The invasive risk model with only HbA1c provides a tool for the targeted selection of patients with the greatest need for intervention.

## SUPPLEMENTARY MATERIAL

eTable 1. Diabetes mellitus model calculations

eTable 2. Characteristics of participants in eight areas in the development cohort (JPHC Diabetes Study)

eTable 3. Characteristics of participants in the development cohort (JPHC Diabetes Study) and the validation cohort (J-ECOH Study)

eTable 4. The results in the development cohort (JPHC Diabetes Study) and in the validation cohort (J-ECOH study) when a family history of diabetes was defined as the presence of diabetes in a family member, regardless of the degree of the relationship

eFigure 1. Association of diabetes mellitus with selected variables in the development cohort (JPHC Diabetes Study)

eFigure 2. Internal-external cross-validation in the JPHC Diabetes Study

eFigure 3. Calibration plots in the development cohort (JPHC Diabetes Study) and in the validation cohort (J-ECOH study) when a family history of diabetes was defined as the presence of diabetes in a family member, regardless of the degree of the relationship

eFigure 4. Calibration plots after intercept adjustment in the validation cohort (J-ECOH Study)

eFigure 5. Receiver operating characteristic curves for the sensitivity analysis in the development cohort (JPHC Diabetes Study) and in the validation cohort (J-ECOH Study)

eFigure 6. Calibration plots for the sensitivity analysis in the validation cohort (J-ECOH Study)
