# Supplementary material for: Development and Validation of Prediction Models for the 5-year Risk of Type 2 Diabetes in a Japanese Population: Japan Public Health Center-based Prospective (JPHC) Diabetes Study
Source: J Epidemiol. 2024 Apr 5;34(4):170–9. doi: 10.2188/jea.JE20220329 (PMC10918338; doi:10.2188/jea.JE20220329)
Supplement: Supplementary file 1 [file je-34-170-s001.zip › JE20230329_eMaterials_final/JE20220329-eTables_eFigures_accepted-34-4-clean.pdf]

eTable 2. Characteristics of participants in eight areas in the development cohort (JPHC Diabetes Study)

Characteristic	Area 1 (n=1,277)	Area 2 (n=1,552)	Area 3 (n=1,624)	Area 4 (n=609)	Area 5 (n=2,740)	Area 6 (n=581)	Area 7 (n=832)	Area 8 (n=1,771)
Age, years	62 (57–66)	62 (57–66)	63 (58–67)	63 (58–67)	62 (55–68)	65 (61–69)	64 (57–70)	65 (59–69)
Women	877 (68.7%)	1,037 (66.8%)	1,138 (70.1%)	407 (66.8%)	1,876 (68.5%)	382 (65.7%)	571 (68.6%)	1,089 (61.5%)
BMI, kg/m^2	23.5 (21.5–25.4)	23.4 (21.5–25.3)	23.2 (21.4–25.2)	24.3 (22.5–26.4)	23.3 (21.2–25.4)	22.5 (20.5–24.5)	23.0 (21.0–25.3)	24.5 (22.6–26.6)
Walking time, hours per day								
<0.5 hours	52 (4.1%)	225 (14.6%)	216 (13.4%)	124 (20.5%)	492 (18.4%)	42 (7.3%)	170 (20.7%)	58 (3.3%)
0.5 hours to <1 hour	130 (10.2%)	452 (29.2%)	308 (19.2%)	227 (37.5%)	651 (24.4%)	73 (12.7%)	239 (29.1%)	242 (13.8%)
1 hour to <2 hours	188 (14.8%)	408 (26.4%)	292 (18.2%)	153 (25.3%)	635 (23.8%)	137 (23.9%)	165 (20.1%)	371 (21.1%)
≥2 hours	904 (71.0%)	461 (29.8%)	792 (49.3%)	101 (16.7%)	893 (33.4%)	322 (56.1%)	247 (30.1%)	1,086 (61.8%)
Family history of diabetes	128 (10.0%)	203 (13.1%)	242 (14.9%)	45 (7.4%)	338 (12.3%)	40 (6.9%)	97 (11.7%)	132 (7.5%)

Family history of diabetes (Not restricted to the first-degree relatives)	158 (12.4%)	240 (15.5%)	282 (17.4%)	61 (10.0%)	420 (15.3%)	50 (8.6%)	142 (17.1%)	172 (9.7%)
SBP, mmHg	130 (119–139)	130 (116–141)	128 (114–138)	130 (120–140)	130 (120–142)	133 (121–147)	132 (120–146)	130 (120–140)
DBP, mmHg	74 (68–81)	78 (70–84)	80 (73–88)	80 (70–84)	78 (70–84)	79 (71– 87)	80 (71–86)	76 (70–80)
HDL, mg/dL	62 (53–72)	59 (49–70)	58 (49–69)	56 (48–66)	53 (45–63)	63 (52–75)	55 (46–65)	55 (47–65)
TC, mg/dL	208 (189–231)	206 (187–227)	209 (188–231)	214 (194–236)	206 (184–229)	208 (187–228)	206 (183–230)	204 (183–227)
FPG, mg/dL	89 (85–95)	95 (90–101)	96 (91–103)	89 (85–95)	93 (88–101)	93 (87–99)	95 (91–100)	94 (89–100)
HbA1c, %	5.6 (5.4–5.8)	5.6 (5.4–5.8)	5.7 (5.5–5.9)	5.5 (5.2–5.7)	5.2 (4.9–5.6)	5.4 (5.2–5.7)	5.2 (5.0–5.4)	5.4 (5.0–5.6)
ALT, IU/L	21 (17–27)	19 (16–25)	19 (15–24)	17 (14–23)	18 (14–23)	17 (13–22)	18 (14–23)	17 (14–23)
AST, IU/L	25 (21–29)	24 (21–28)	22 (19–26)	20 (17–23)	23 (19–27)	23 (20–27)	22 (19–26)	21 (18–24)
GGT, IU/L	23 (17–35)	20 (14–33)	21 (16–32)	26 (19–40)	17 (13–26)	21 (16–30)	17 (12–28)	26(19–39)
eGFR, mL/min/1.73 m^2	68.5 (62.8–78.6)	78.2 (65.7–98.6)	73.2 (63.1–76.7)	66.0 (58.8–76.0)	74.1 (63.6–82.0)	84.7 (74.4–93.6)	69.1 (59.9–81.1)	66.4 (60.1–76.3)

5-year outcome	79	91	80	40	248	50	50	69

ALT, alanine aminotransferase; AST, aspartate aminotransferase; BMI, body mass index; DBP, diastolic blood pressure; eGFR, estimated glomerular filtration rate; FPG, fasting plasma glucose; GGT, γ-glutamyl transferase; HbA1c, glycated hemoglobin; HDL, high-density lipoprotein; SBP, systolic blood pressure; TC, total cholesterol.

Characteristics were collected at baseline.

Continuous variables are medians (interquartile ranges), and categorical variables are numbers (percentages).

eTable 3. Characteristics of participants in the development cohort (JPHC Diabetes Study) and the validation cohort (J-ECOH Study)

	JPHC Diabetes Study			J-ECOH Study	
Characteristic	Participants in the final analysis (n= 10,986)	Participants not in the final analysis (n= 14,596)	Characteristic	Participants in the final analysis (n= 11,345)	Participants not in the final analysis (n=5,819)
Age, years	63 (57–67)	63 (56–68)	Age, years	51 (48–54)	58 (53–62)
Women	7,377 (67.1%)	9,323 (63.9%)	Women	1,773 (15.6 %)	1,044 (17.9 %)
BMI, kg/m^2	23.5 (21.5–25. 6)	23.4 (21.4–25. 6)	BMI, kg/m^2	23.2 (21.4–25.3)	23.2 (21.4–25.2)
Walking time, hours per day			Leisure-time physical activity, minutes per month	0 (0–84)	0 (0–98)
<0.5 hours	1,379 (12. 6%)	2,441 (16.7%)			
0.5 hours to <1 hour	2,322 (21.1%)	3,624 (24.8%)			
1 hour to <2 hours	2,349 (21.4%)	2,898 (19.9%)			
≥2 hours	4,806 (43.7%)	5,465 (37.4%)			
Family history of diabetes	1,225 (11.2%)	1,692 (11.6%)	Family history of diabetes	1,996 (17.6%)	894 (15.4 %)
SBP, mm Hg	130 (119–140)	130 (119–141)	SBP, mm Hg	122 (113–130)	124 (115–133)
DBP, mm Hg	78 (70–84)	78 (70–84)	DBP, mm Hg	79 (72–84)	79 (73–85)
HDL, mg/dL	57 (48–67)	58 (48–68)	HDL, mg/dL	55 (46–65)	55 (47–67)
TC, mg/dL	207 (186–230)	207 (184–229)	TC, mg/dL	201 (181–221)	200 (180–222)

FPG, mg/dL	93 (88–100)	95 (89–102)	FPG, mg/dL	98 (92–105)	99 (93–106)
HbA1c, %	5.5 (5.1–5.7)	5.5 (5.2–5.8)	HbA1c, %	5.5 (5.3–5.7)	5.6 (5.4–5.8)
ALT, IU/L	18 (15–24)	19 (15–25)	ALT, IU/L	21 (16–29)	20 (15–27)
AST, IU/L	22 (19–27)	23 (19–27)	AST, IU/L	21 (18–26)	21 (18–26)
GGT, IU/L	21 (15–33)	22 (15–35)	GGT, IU/L	30 (20–51)	30 (20–50)
eGFR, mL/min/1.73 m^2	73.8 (63.4–82.5)	74.7 (63.9–88.1)	eGFR, mL/min/1.73 m^2	78.8 (69.7–89.4)	76.5 (66.9–87.7)

ALT, alanine aminotransferase; AST, aspartate aminotransferase; BMI, body mass index; DBP, diastolic blood pressure; eGFR, estimated glomerular filtration rate; FPG, fasting plasma glucose; GGT, γ-glutamyl transferase; HbA1c, glycated hemoglobin; HDL, high-density lipoprotein; SBP, systolic blood pressure; TC, total cholesterol.

Characteristics were collected at baseline.

Continuous variables are medians (interquartile ranges), and categorical variables are numbers (percentages).

eTable 4. The results in the development cohort (JPHC Diabetes Study) and in the validation cohort (J-ECOH study) when a family history of diabetes was defined as the presence of diabetes in a family member, regardless of the degree of the relationship

Characteristics of participants in the development cohort

Characteristic	Development cohort (n=10,986)	
	Value	Missing values, n (%)
Family history of diabetes	1,525 (13. 9%)	18 (0.2)

Distribution of study variables by DM status in the development cohort

Characteristics	Participants without incident DM (n=10,279)	Participants with incident DM (n=707)	Odds ratio (95% CI)			
			Univariate	Model 1	Model 2	Model 3
Family history of diabetes (%)						
Yes	1,363 (89%)	162 (11%)	1.94 (1.62–2.34)	2.01 (1.67–2.43)	1.57 (1.29–1.92)	1.47 (1.18–1.84)
No	8,916 (94%)	545 (6%)	1 (ref.)	1 (ref.)	1 (ref.)	1 (ref.)

The area under the receiver operating characteristic curve (AUC) in the development cohort

	Model1	Model2	Model3
AUC	0.641	0.785	0.846

The area under the receiver operating characteristic curve (AUC) in the validation cohort

	Model1	Model2	Model3
AUC	0.695	0.831	0.875

AUC, the area under the receiver operating characteristic (ROC) curves; CI, confidence interval; DM, diabetes mellitus.

eFigure 1. Association of diabetes mellitus with selected variables in the development cohort (JPHC Diabetes Study)

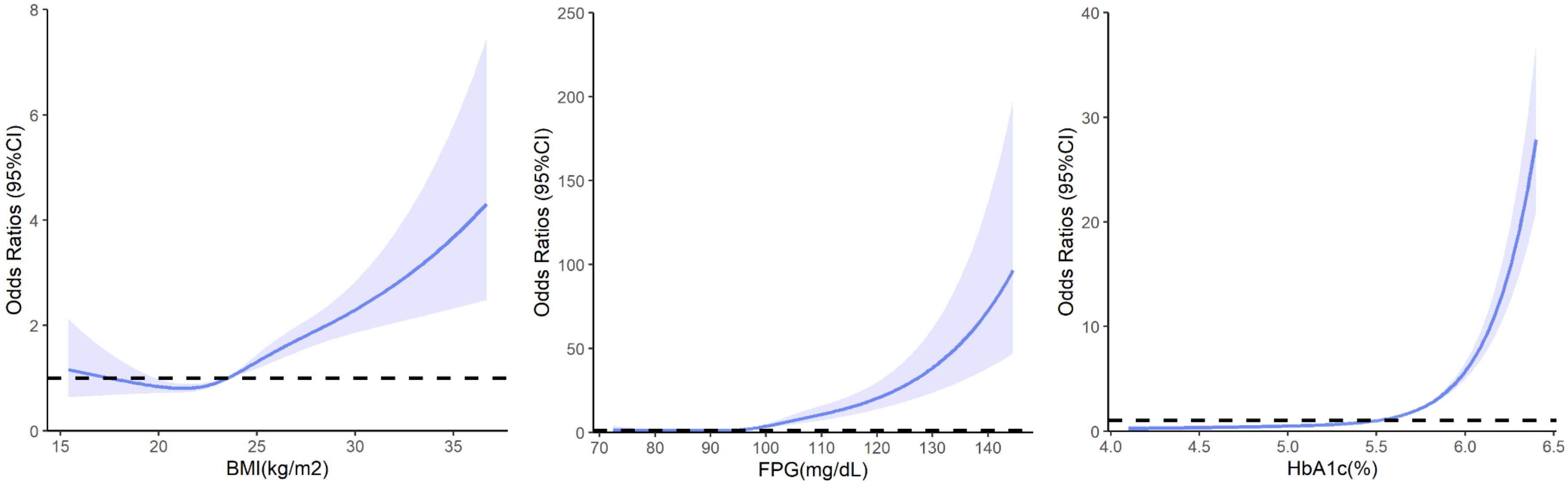

BMI, body mass index; FPG, fasting plasma glucose; HbA1c, glycated hemoglobin.
Odds ratios were estimated using logistic regression models for each univariable after multiple imputations.
Odds ratios are indicated by solid lines and 95% confidence intervals by shaded areas.
The reference point was placed where the odds ratio is equal to 1. Knots were placed at the 10th, 50th and 90th centiles for HbA1c, at the 5th, 35th, 65th, and 95th centiles for BMI, and at the 5th, 27.5th, 50th, 72.5th, and 95th centiles for FPG.

eFigure 2. Internal-external cross-validation in the JPHC Diabetes Study

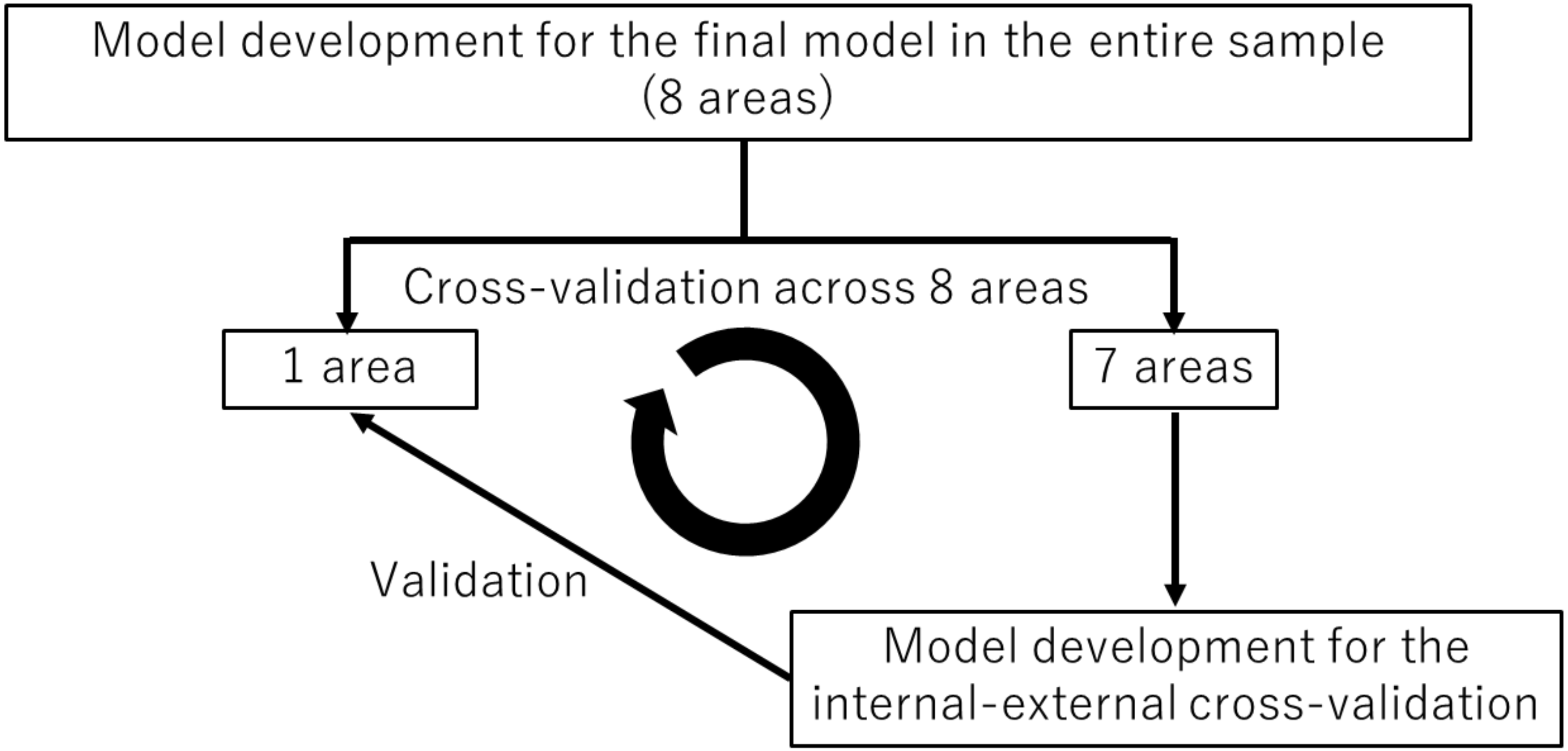

In the internal-external cross-validation procedure, the model development was performed in 7 areas by sequentially dropping one area at a time, and model discrimination was examined in the omitted area.

eFigure 3. Calibration plots in the development cohort (JPHC Diabetes Study) and in the validation cohort (J-ECOH study) when a family history of diabetes was defined as the presence of diabetes in a family member, regardless of the degree of the relationship

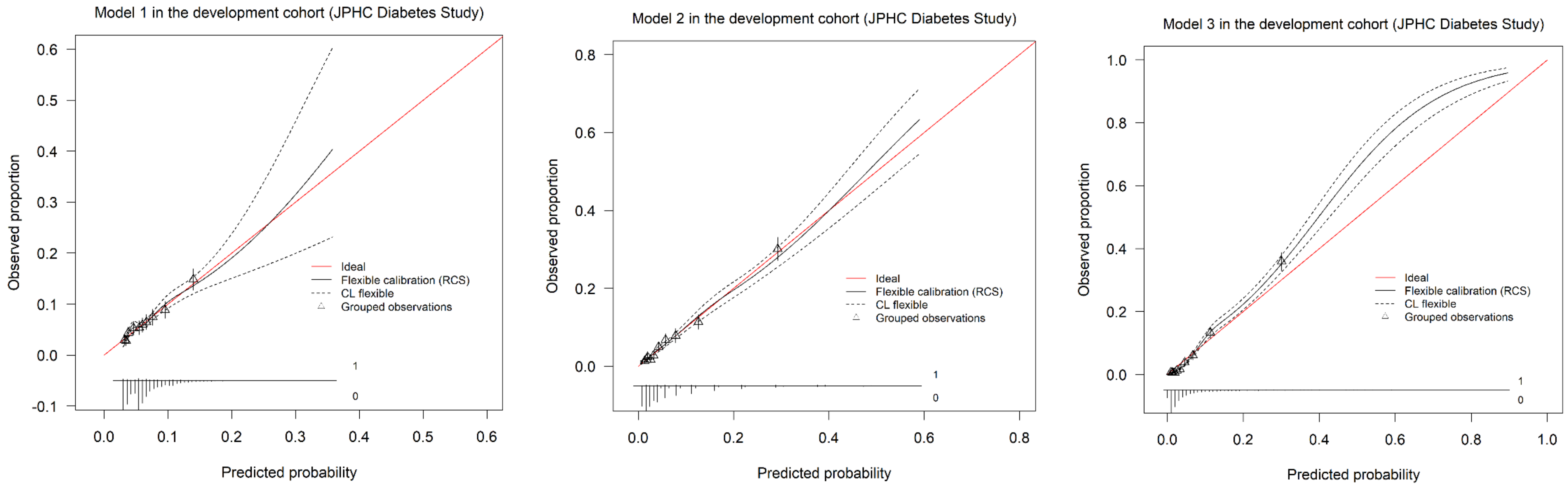

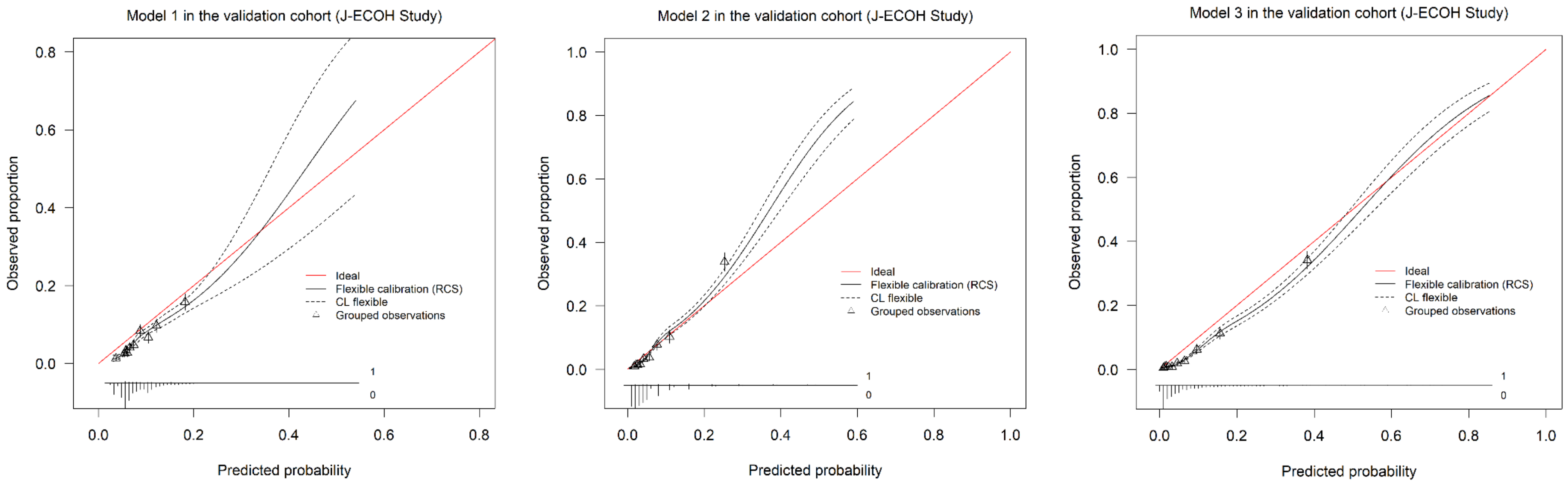

Ideal: ideal line for the prediction model. Flexible calibration (RCS): "RCS" generates a flexible calibration curve based on restricted cubic splines. CL flexible: 95% confidence limits for the flexible calibration curve with dashed lines. Grouped observations: mean predicted probability and observed proportion of diabetes incidence in each of the deciles (ten groups of equal size).

eFigure 4. Calibration plots after intercept adjustment in the validation cohort (J-ECOH Study)

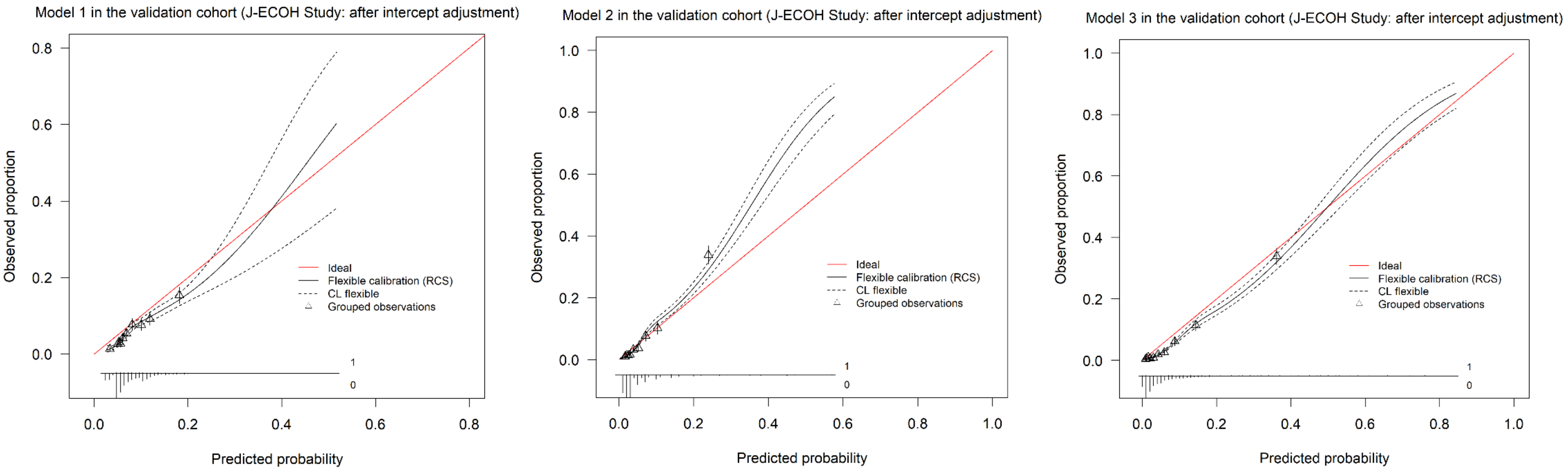

Ideal: ideal line for the prediction model. Flexible calibration (RCS): "RCS" generates a flexible calibration curve based on restricted cubic splines. CL flexible: 95% confidence limits for the flexible calibration curve with dashed lines. Grouped observations: mean predicted probability and observed proportion of diabetes incidence in each of the deciles (ten groups of equal size).

eFigure 5. Receiver operating characteristic curves for the sensitivity analysis in the development cohort (JPHC Diabetes Study) and in the validation cohort (J-ECOH Study)

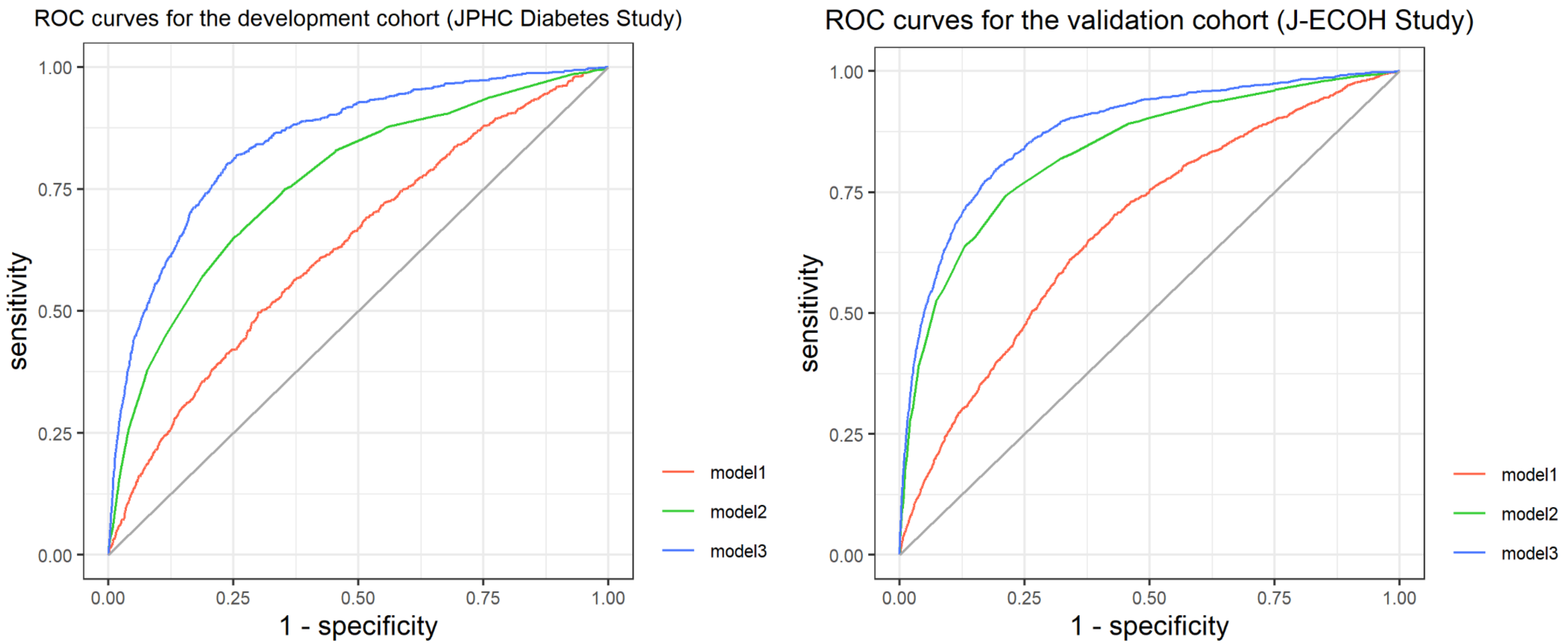

AUC, the area under the receiver operating characteristic (ROC) curves.
A sensitivity analysis among 23,957 participants including those who did not visit the 5-year follow-up survey in the development cohort (JPHC Diabetes Study).
C-statistic (AUC): Model 1 = 0.631, Model 2 = 0.764, Model 3 = 0.848.

A sensitivity analysis among 17,164 participants including those who did not visit the 5-year follow-up survey in the validation cohort (J-ECOH Study).
C-statistic (AUC): Model 1 = 0.676, Model 2 = 0.834, Model 3 = 0.874.

eFigure 6. Calibration plots for the sensitivity analysis in the validation cohort (J-ECOH Study)

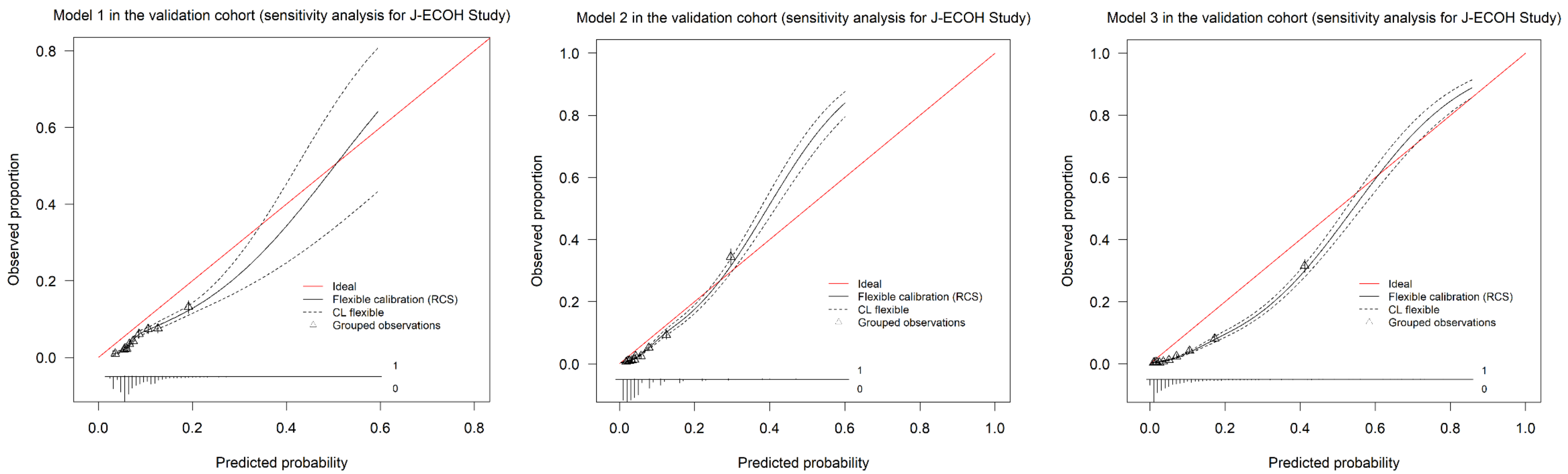

A sensitivity analysis among 17,164 participants including those who did not visit the 5-year follow-up survey in the validation cohort (J-ECOH Study). Calibration plots were created to graphically assess the agreement of the mean observed risk with the mean predicted risk according to the deciles of the predicted risk. Ideal: ideal line for the prediction model. Flexible calibration (RCS): "RCS" generates a flexible calibration curve based on restricted cubic splines. CL flexible: 95% confidence limits for the flexible calibration curve with dashed lines. Grouped observations: mean predicted probability and observed proportion of diabetes incidence in each of the deciles (ten groups of equal size).
